# 366. Association Between Gut Microbiota and Health-Related Quality of Life in Patients with Recurrent *Clostridioides difficile* Infection: Results from the PUNCH CD3 Clinical Trial

**DOI:** 10.1093/ofid/ofad500.436

**Published:** 2023-11-27

**Authors:** Paul Feuerstadt, Ken Blount, Amy Guo, Min Yang, Viviana García-Horton, Mirko Fillbrunn, Glenn S Tillotson, Yipeng Gao, Erik R Dubberke, Kevin W Garey

**Affiliations:** Yale University School of Medicine/PACT-Gastroenterology Center, Westport, Connecticut; Rebiotix, Inc., Roseville, Minnesota; Ferring Pharmaceuticals, parsippany, New Jersey; Analysis Group, Inc., Boston, Massachusetts; Analysis Group, New York City, New York; Analysis Group, Inc., Boston, Massachusetts; GST Micro LLC, NORTH, Virginia; Analysis Group, Inc., Boston, Massachusetts; Washington University, Saint Louis, Missouri; University of Houston, Houston, TX

## Abstract

**Background:**

Recurrence of *Clostridioides difficile* infection (rCDI) substantially diminishes patients’ health-related quality of life (HRQL). The gut microbiome is known to play an important role in rCDI, but little is known about its relationship with HRQL. In this study, we assessed the relationship between the gut microbiome and HRQL in patients with rCDI from REBYOTA’s (fecal microbiota, live-jslm [RBL]) phase 3 PUNCH CD3 trial (NCT03244644).

**Methods:**

HRQL and relative abundance (i.e., the % of one microbiota relative to all other analyzed) of gut microbiota at the class level were collected at baseline and weeks 1, 4, and 8. The Microbiome Health Index (MHI; Blount et al, 2022), defined as (Bacteroidia + Clostridia) / (Gammaproteobacteria + Bacilli), was analyzed using log transformation and as a binary indicator with MHI > 7.2 indicating a healthy microbiome. HRQL was measured using the disease-specific *C. difficile* Quality of Life Survey (Cdiff32) via its total score and physical, mental, and social domain scores (0–100 range, 100 best).

Correlations between microbiota data (Bacteroidia, Clostridia, Gammaproteobacteria, Bacilli, and log MHI) and Cdiff32 scores were estimated. Associations of Cdiff32 scores with microbiota data and MHI > 7.2 were estimated using mixed effects analyses adjusted for baseline patient characteristics. Analyses used all available data (baseline, weeks 1, 4, 8). P-values were adjusted for multiple testing via the Benjamini-Hochberg procedure; correlations were interpreted as moderate if ≥ 0.3 and large if ≥ 0.5 (Cohen, 1988).

**Results:**

A total of 176 out of 262 (67.2%) patients had Cdiff32 and microbiota data (119 RBL, 57 Placebo) in PUNCH CD3. All four Cdiff32 scores correlated positively with log MHI (∼0.6), Clostridia (∼0.5) and Bacteroidia (∼0.3), and negatively with Gammaproteobacteria (∼−0.5) and Bacilli (∼−0.3) (**Table 1**). Significant associations of microbial relative abundances and log MHI with all Cdiff32 scores were found in mixed effects analyses (all p< 0.01) (**Table 2**). MHI > 7.2 (healthy microbiome) was associated with an improvement of 14.2 to 18.4 points in Cdiff32 scores (vs. MHI ≤ 7.2).

**Figure:**
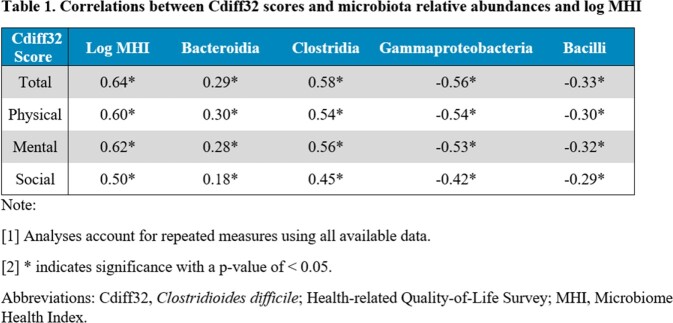

**Figure:**
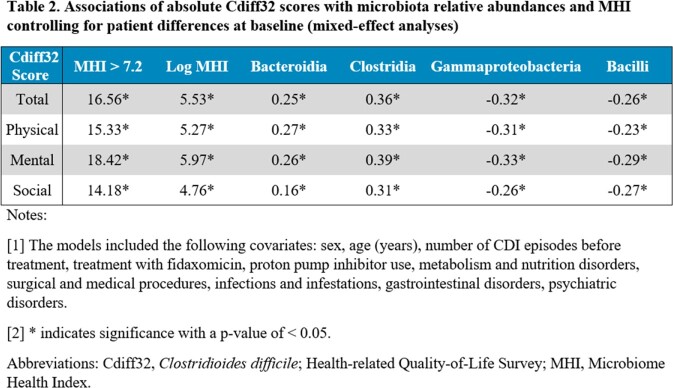

**Conclusion:**

Our study found moderate to large and statistically significant associations between a healthy gut microbiome and improved HRQL in patients with rCDI.

**Disclosures:**

**Paul Feuerstadt, MD, FACG, AGAF**, Ferring/Rebiotix Pharmaceuticals: Advisor/Consultant|Ferring/Rebiotix Pharmaceuticals: Grant/Research Support|Merck and Co.: Advisor/Consultant|Seres Therapeutics: Advisor/Consultant|Seres Therapeutics: Grant/Research Support|Takeda Pharmaceuticals: Advisor/Consultant **Ken Blount, PhD**, Ferring Pharmaceuticals: Employee **Amy Guo, PhD**, Ferring Pharmaceuticals: Employee **Min Yang, MD, PhD**, Analysis Group, Inc.: I am an employee of Analysis Group, Inc., which has received consulting fees from Ferring for the conduct of this study. **Viviana García-Horton, PhD**, Analysis Group, Inc.: I am an employee of Analysis Group, Inc., which has received consulting fees from Ferring for the conduct of this study. **Mirko Fillbrunn, PhD**, Analysis Group, Inc.: I am an employee of Analysis Group, Inc., which has received consulting fees from Ferring for the conduct of this study. **Glenn S. Tillotson, PhD**, Dynavax: Advisor/Consultant|Ferring Pharmaceuticals: Advisor/Consultant|Peggy Lillis Foundation: Honoraria|Spero Therapeutics: Advisor/Consultant **Yipeng Gao, PhD**, Merck and Co.: Grant/Research Support **Erik R. Dubberke, MD, MSPH**, Abbott: Advisor/Consultant|AstraZeneca: Advisor/Consultant|Ferring Pharmaceuticals: Advisor/Consultant|Ferring Pharmaceuticals: Grant/Research Support|Merck and Co.: Advisor/Consultant|Pfizer: Advisor/Consultant|Pfizer: Grant/Research Support|Seres Therapeutics: Advisor/Consultant|Summit: Advisor/Consultant|Theriva Biologics: Grant/Research Support **Kevin W. Garey, PharmD, MS**, Acurx: Grant/Research Support|Ferring: Advisor/Consultant|Paratek: Grant/Research Support

